# Proteomic adaptation of the hypervirulent pandemic *Vibrio parahaemolyticus* ST36 lineage to salinity and temperature stress

**DOI:** 10.3389/fmicb.2026.1947974

**Published:** 2026-10-02

**Authors:** Eloisa González-Vidal, Sara Eslava-Alcón, Estefanía Jurado-Tarifa, Inmaculada Guerrero-Lozano, MaCarmen Durán-Ruiz, Manuel A. Rodriguez-Iglesias

**Affiliations:** 1 Biomedicine, Biotechnology and Public Health Department, University of Cadiz, Cádiz, Spain; 2 Biomedical Research and Innovation Institute of Cadiz (INiBICA), Cádiz, Spain; 3 Service of Microbiology, Puerta del Mar University Hospital, Cádiz, Spain

**Keywords:** environmental adaptation, foodborne diseases, marine pathogens, proteomic profile, salinity stress, thermal stress, *Vibrio parahaemolyticus*, virulence factors

## Abstract

*Vibrio parahaemolyticus* is a halophilic Gram-negative bacterium widely distributed in marine and estuarine ecosystems, and a leading cause of seafood-borne gastroenteritis worldwide. Among its genetic lineages, sequence type 36 (ST36) has emerged as a pandemic clone with remarkable environmental persistence and global dissemination. Its pathogenicity is closely associated with environmental conditions, particularly salinity and temperature, which modulate the expression of virulence factors, stress-response proteins, and metabolic pathways. In this study, we investigated the differential protein expression profiles of *Vibrio parahaemolyticus* under controlled salinity (1 and 3%) and temperature (20 °C, 31 °C, and 37 °C) using a label free-quantitative (LFQ) proteomic approach. Our results demonstrate that the *Vibrio parahaemolyticus* proteome is dynamically and non-linearly modulated by specific combinations of salt and heat rather than a simple additive effect of these factors. Temperature-salinity interactions drove most expression changes (2,144 proteins), with temperature acting as the primary driver (1,302 proteins). Proteome changes peaked mostly at 31 °C for salinity variations and at 3% salinity for temperature shifts, each affecting ~80% of proteins, though specific responses ultimately depended on the nature of individual proteins. Functionally, protein changes were representative of a shift from relatively balanced cellular metabolism at mild stress conditions toward respiratory remodeling, central-carbon reorganization, ATP production, and redox defense under elevated salinity and temperature. Additionally, virulence-, toxin-, motility-, chemotaxis-, and defense-associated proteins were distributed across several trend clusters. Overall, the proteomic patterns detected in *Vibrio parahaemolyticus* in response to temperature and salinity fluctuations reveal a coordinated regulation of virulence, stress-response, and metabolic proteins that underpins its ecological fitness. Beyond these biological insights, the proteomic workflow employed provides a robust methodological framework for studying bacterial stress adaptation. Ultimately, these findings deepen our understanding of the pathogen dynamics under climate change and for improving seafood safety and risk management. Future research is warranted to experimentally validate the molecular pathways identified and confirm their functional roles in adaptation.

## 1 Introduction

*Vibrio parahaemolyticus* is a halophilic Gram-negative bacterium widely distributed in marine and estuarine ecosystems and is recognized for its pathogenic potential in humans through the consumption of raw or undercooked seafood. This microorganism is one of the leading causes of acute bacterial gastroenteritis associated with seafood products worldwide (Su and Liu, 2007; Baker-Austin et al., 2017). In recent decades, several studies have reported an increase in epidemic outbreaks caused by *Vibrio parahaemolyticus* in coastal regions of Asia, the Americas, and Europe, a phenomenon linked to the globalization of seafood trade and rising ocean temperatures (Baker-Austin et al., 2017; Letchumanan et al., 2014; Park et al., 2018).

Among the numerous lineages of *Vibrio parahaemolyticus*, sequence type 36 (ST36) has emerged as one of the most clinically significant pandemic clones worldwide. Originally associated with the Pacific Northwest of North America, ST36 has expanded geographically during the last two decades and has been responsible for numerous seafood-associated outbreaks in North America and Europe (Abanto et al., 2020). Its remarkable environmental fitness, ability to colonize diverse marine ecosystems, and enhanced virulence profile have raised considerable concern from both public health and food safety perspectives. Increasing evidence suggests that climate-driven changes in seawater temperature and salinity may facilitate the persistence and geographic expansion of this lineage, emphasizing the need to understand the molecular mechanisms underlying its environmental adaptation (Baker-Austin et al., 2017).

Phylogenetically, *Vibrio parahaemolyticus* belongs to the genus *Vibrio*, which includes highly versatile bacterial species capable of adapting to fluctuating environmental conditions through genomic plasticity and efficient stress-response mechanisms (Kita-Tsukamoto et al., 1993). This adaptive capacity is primarily related to the bacterium’s ability to regulate gene expression in response to key factors such as temperature and salinity, which play a decisive role in its ecology, survival, and virulence (Raghunath, 2014; Wong et al., 2000).

Regarding pathogenicity, the best-characterized virulence factors are the thermostable hemolysins TDH and TRH, whose expression levels vary with temperature and are directly associated with the severity of gastrointestinal infections (Su and Liu, 2007; Zhang and Orth, 2013). Additionally, numerous proteins involved in thermal, osmotic, and environmental stress responses have been shown to play a role in the adaptation of *Vibrio parahaemolyticus* to shifting environmental conditions, supporting its persistence and infectious potential under adverse scenarios (Raghunath, 2014; Caburlotto et al., 2010).

Recent studies have demonstrated that both temperature and salinity can significantly modulate the expression of these virulence and adaptative genes. Extreme or fluctuating conditions trigger the activation of transport systems, the accumulation of compatible solutes, the expression of stress proteins, and the secretion of virulence factors, all critical survival mechanisms against osmotic and thermal stress (Raghunath, 2014; Caburlotto et al., 2010; Milton, 2006). This molecular plasticity enables *Vibrio parahaemolyticus* to adapt, persist, and colonize diverse marine environments, potentially enhancing its virulence under favorable conditions (Baker-Austin et al., 2017; Yildiz and Visick, 2009).

The present study provides the first integrated proteomic characterization of the *Vibrio parahaemolyticus* response to the combined effects of salinity and temperature stress under environmentally relevant conditions. Unlike previous approaches, which mainly focused on individual stressors, transcriptomic analyses, or host-associated conditions, this work simultaneously evaluates the impact of different combinations of salinity (1 and 3%) and temperature (20 °C, 31 °C, and 37 °C) on the global proteomic profile of *Vibrio parahaemolyticus*. By applying a label-free quantitative (LFQ) proteomics analysis, we identified differential expression patterns in proteins involved in virulence, osmotic adaptation, heat-shock response, oxidative stress defense, metabolism, and cell signaling. Importantly, this study reveals for the first time a coordinated proteomic remodeling dependent on the interaction between salinity and temperature. Indeed, more than 82% of protein changes were driven by temperature-salinity interactions rather than the single factors alone, although the specific response trends were ultimately associated with each individual protein. Ultimately, these findings expand our understanding of the *Vibrio* stress response under fluctuating environmental conditions by highlighting key differentially expressed proteins that may be relevant to future sanitary surveillance and control strategies in marine and seafood-associated environments.

## 2 Materials and methods

### 2.1 Bacterial strain

A comprehensive proteomic analysis was performed on an environmental strain of *Vibrio parahaemolyticus* belonging to the ST36 clone, a hypervirulent lineage previously associated with international outbreaks (Abanto et al., 2020).

The ST36 strain analyzed was isolated from a sediment sample collected by our research in October 2021 group at Bajo de Guia (Guadalquivir River estuary, Sanlúcar de Barrameda, South Western Spain), during an environmental surveillance study. This area represents a transitional ecosystem between freshwater and marine environments, characterized by constant fluctuations in salinity, temperature, and other physicochemical factors. The isolation procedure from the sediment was plating in saline alkaline peptone broth with subsequent isolation on Chromid Vibrio chromogenic plates (BioMerieux). Identification was performed by MALDI-TOF (Bruker Biotyper). The strain was sequenced using MySeq sequencer (Illumina) and the biosample sequence is deposited in GenBank (SAMN35217047).

### 2.2 *Vibrio parahaemolyticus* ST36 strain culture

Isolated *Vibrio parahaemolyticus* ST36 strain was initially cultured on blood agar and incubated for 24 h at 36 °C. Bacterial growth was subsequently resuspended in 2 mL of sterile saline solution and adjusted to a turbidity equivalent to a 0.5 McFarland standard. For each experimental condition, tubes containing 5 mL of alkaline peptone water adjusted to the corresponding NaCl concentration were inoculated with 200 μL of the standardized bacterial suspension.

Cultures were established under two salinity conditions (1 and 3% NaCl) and incubated at three different temperatures (20 °C, 31 °C, and 37 °C), resulting in six experimental conditions (1% NaCl/20 °C, 1% NaCl/31 °C, 1% NaCl/37 °C, 3% NaCl/20 °C, 3% NaCl/31 °C, and 3% NaCl/37 °C). The 3% NaCl and 20 °C parameters mirrored the environmental conditions recorded during the collection of the sediment containing the ST36 strain (3.33% salinity, 21.4 °C surface water temperature). Additionally, incubation at 1% NaCl represented a lower-salinity regime, while 31 °C and 37 °C provided progressively higher-temperature conditions. This design allowed the evaluation of the isolate ST36 proteomic response across defined osmotic and thermal gradients, relative to baseline conditions encountered in its native environment.

Three independent cultures were prepared for each of the six experimental conditions and incubated for 18 h at the corresponding temperature (18 samples in total). After incubation, 2 mL of each bacterial culture were transferred to sterile microcentrifuge tubes and centrifuged at 10,000 × g for 3 min to harvest the bacterial cells. The supernatants were discarded, and the resulting bacterial pellets were retained for subsequent protein extraction.

### 2.3 Sample preparation for proteomic analysis

Protein extraction and digestion was carried out following a standardized protocol to ensure reproducibility. First, *Vibrio parahaemolyticus* pellets were lysed by bead beating in 8 M urea and 10 mM Tris (pH 8.0) using three 30-s cycles with 1-min rests on ice. Lysates were centrifuged at ≥13,000 × g for 5 min, and supernatants were collected. Proteins were precipitated with 3 mL of cold acetone, then resuspended in 100 μL of 8 M urea and quantified using the Qubit Protein Assay Kit (Thermo Fisher). A 1:10 dilution was used for quantification. For digestion, 50 μg of protein per sample was reduced with 10 mM DTT for 30 min at room temperature and alkylated with 50 mM IAA in the dark for another 30 min. Samples were adjusted to 70% acetonitrile and incubated with magnetic beads (20 mg/mL) at a 4:1 bead-to-protein ratio. After two binding cycles, beads were washed twice with 95% acetonitrile and twice with 70% ethanol, then resuspended in 300 μL of 50 mM ammonium bicarbonate (ABC). Proteolytic digestion was carried out using a Trypsin/Lys-C mix (Promega, 0.2 μg/μL) added at a 1:50 enzyme-to-protein mass ratio. The digestion was performed overnight at 37 °C under gentle agitation to ensure efficient and complete peptide cleavage. Digestion was stopped by acidification with 5% TFA (pH 1–2), and peptides were purified using the EvoTip protocol (Evosep) for minimal sample loss. Eluates were dried in a SpeedVac (Thermo Fisher, 3.5 h) and stored at −80 °C until LC–MS/MS analysis.

### 2.4 LC–MS/MS analysis

Peptide separation and mass spectrometry (MS) analysis were performed using a timsTOF Pro quadrupole time-of-flight (QTOF) mass spectrometer (Bruker, Billerica, MA, United States) integrated with a nanoElute2 UHPLC system (Bruker). Chromatographic resolution was achieved using a C18 PepSep column (150 mm × 75 μm, 1.9 μm particle size, 120 Å pore size, Bruker Daltonics, Bremen, Germany). Dried peptide samples were reconstituted prior LC–MS/MS analysis in 30 μL of aqueous solution containing 0.1% formic acid, to a final concentration of 100 ng/μl, injecting 100 ng (1 μL) of peptides per sample. Peptides were separated over a 30-min gradient ranging from 2 to 35% of solvent B (acetonitrile with 0.1% formic acid), followed by a rapid increase to 95% B over 3 min and a 7-min high-strength wash at 95% B. The flow rate was set at 300 nL/min, with the column maintained at 40 °C. Ionization of peptides was carried out using a CaptiveSpray nanoelectrospray ion source (Bruker) at an applied voltage of 1,500 V. Mass spectrometric data were acquired in diaPASEF mode, in a data independent acquisition (DIA) analysis, as previously described by Arenas-De Larriva et al. (2022).

### 2.5 Protein identification and data processing

Raw diaPASEF files were processed using DIA-NN v1.8.1 (Demichev et al., 2018), a library-free software optimized for data-independent acquisition (DIA) analysis. Protein identification was achieved by searching against the *Vibrio parahaemolyticus UniProtKB/*SwissProt-reviewed FASTA database (downloaded on October, 2023). No contaminant FASTA database was included in the analysis. The searching process included deep learning–based predictions for MS/MS spectra, retention time, and ion mobility. Also, up to two missed cleavages and a maximum of two variable modifications per peptide were allowed. Carbamidomethylation of cysteine was set as a fixed modification, whereas methionine oxidation and N-terminal acetylation were considered variable modifications. Identifications were filtered using a precursor-level *q*-value threshold of 0.01, corresponding to a 1% false discovery rate.

The resulting LFQ protein intensity values matrix was analyzed in RStudio (version 2024.12.1 + 563) using a standardized workflow. Protein intensity values were log₂-transformed, and proteins detected in at least 70% of the samples were retained for downstream analysis. Missing values were imputed by random sampling from a normal distribution using a width of 0.5 and a downshift of 1.8 standard deviations. Exploratory data analysis was performed on the processed matrix using principal component analysis (PCA) to assess sample relationships and overall data structure. Differential protein expression was evaluated using the limma package by fitting protein-wise linear models and defining the corresponding statistical contrasts.

Functional enrichment analysis was carried out with proteins meeting the filtering and statistical criteria using python with the Julius AI online software.^1^ Both, protein functions and subcellular locations were defined based on Uniprot Protein description and KEGG Pathway information.

### 2.6 Statistical analysis

The log_2_ intensity values of proteins that passed the 70% filter were analyzed across a 2 × 3 factorial experimental design: salinity (1% S, 3% S), and temperature (20 °C, 31 °C, 37 °C), including 3 technical replicates per condition (18 measurements per protein). For each protein, a two-way ANOVA (type II sums of squares) test was applied to assess the main effects of salinity, temperature and the interaction of both factors. Nominal *p*-values were adjusted for multiple testing using the Benjamini–Hochberg false discovery rate procedure, separately for the salinity, temperature, and salinity × temperature interaction. For each ANOVA term, F-statistics and nominal *p*-values were calculated. Proteins with an adjusted *p*-value, or FDR, below 0.05 were considered statistically significant. Proteins were considered differentially expressed when they met both a nominal *p*-value threshold of ≤ 0.01 and an absolute log₂ fold-change threshold of ≥ 1.

Proteins were classified into four response categories based on ANOVA terms: saline-only or temperature-only (sole main effect), additive (both main effects without interaction), or condition-dependent, for proteins with a significant salinity × temperature interaction. For the last ones, follow-up simple-effect contrasts were conducted using the pooled residual variance, with 12 residual degrees of freedom. These included temperature comparisons within each salinity level (1% S, 3% S): 37 °C *vs* 20 °C, 37 °C *vs* 31 °C, and 31 °C *vs* 20 °C, and salinity comparisons, 3% *vs* 1%, at each temperature. Effect sizes were calculated as mean abundance differences, and contrast-specific *p*-values were FDR-adjusted (Benjamini–Hochberg) across all 2,620 proteins per contrast.

Principal component analysis (PCA) was performed using the mean-centered protein intensity values across 18 samples to assess sample reproducibility and global separation among salinity–temperature conditions. To evaluate proteome-wide trends across all six conditions, protein mean abundances were averaged per condition and z-score standardized. Next, proteins were categorized using k-means clustering (k = 6,50 random initializations, fixed seed) to visualize major salinity- and temperature-dependent expression patterns. All analyses and visualizations were implemented in Python using pandas, SciPy, statsmodels, scikit-learn, and matplotlib, via Julius AI online software (see text footnote 1, respectively).

## 3 Results

### 3.1 Differential proteomic response of *Vibrio parahaemolyticus* to salinity and temperature stress

To evaluate the adaptive response of *Vibrio parahaemolyticus* across different environmental salinity (1% S and 3% S) and temperature (20 °C, 31 °C, and 37 °C) growth conditions, a LFQ proteomic analysis was carried out (Figure 1). In total, 2,620 proteins were identified across all 6 conditions in at least 70% of all 18 samples analyzed. Next, the individual effects of salinity and temperature, as well as their interaction, were evaluated using a two-way ANOVA, and the results were adjusted to control false discovery rates (FDR < 0.05). Detailed information regarding protein identification, LFQ intensities, fold changes, and statistical significance is provided in Supplementary Table S1.

**Figure 1:**
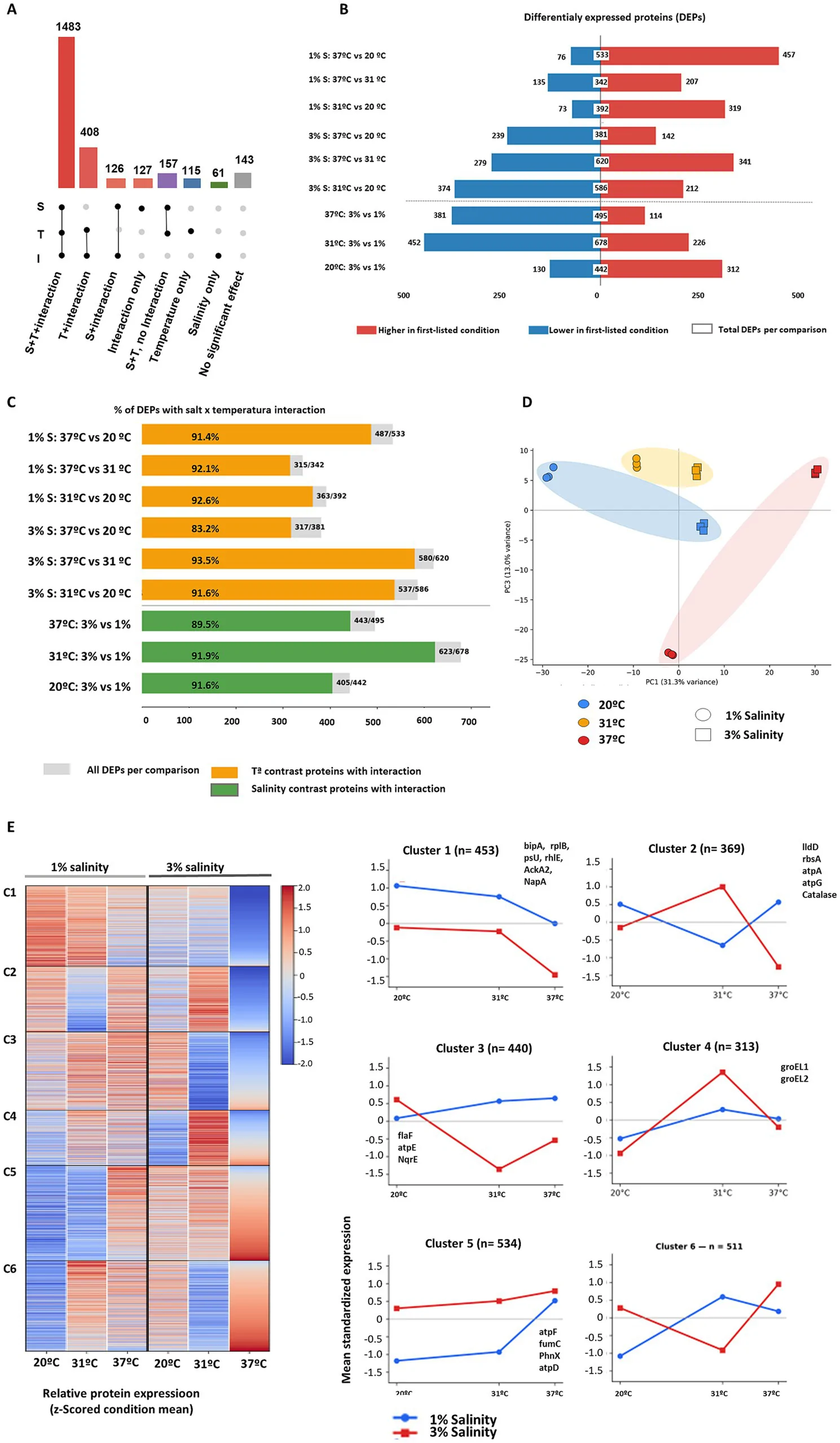
Differential proteomic profiling of *Vibrio parahaemolyticus* under salinity and temperature stress conditions. **(A)** Intersection plot, summarizing the ANOVA results, indicating the number of proteins affected mainly by Salinity (S), Temperature (T), or by S + T interaction. Each bar represents a mutually exclusive protein category, where black dots mean indicates significant ANOVA factors. The main-effect totals can be calculated by adding every bar that contains a dot. So, the salinity main effect (1827) proteins while T affects to 2,163 proteins. **(B)** Diverging bar chart showing the number of proteins up- (red) and down-regulated (blue) across pairwise comparisons. Thresholds are FDR < 0.05 and |logFC| ≥ 1. **(C)** Graphical integration of pairwise DEPs with the formal ANOVA interaction results. Colored bar segments indicate the number of DEPs in each pairwise comparison that also have a significant salinity × temperature interaction. **(D)** Principal component analysis (PCA) illustrating sample clustering and separation across salinity and temperature conditions (*n* = 3 biological replicates/condition). **(E)** Expression trend clustering heatmap of significantly regulated proteins across experimental conditions. The line plots on the right show the average expression trends for proteins in each cluster, comparing 1% salinity with 3% salinity across 20 °C, 31 °C, and 37 °C.

According to our results, temperature was the predominant factor, affecting 2,163 out of the 2,620 identified proteins, whereas salinity on its own affected 1827 proteins (Figure 1A). Specifically, 115 protein changes were exclusively attributed to temperature and 61 exclusively to salinity. As expected, most expression changes were driven by temperature–salinity interactions, with 2,144 proteins affected jointly by both factors, rather than by temperature or salinity factors alone. Among these 2,144 interactively-affected proteins, temperature was the primary driver for most changes (1,302 proteins), although nearly two in five proteins were more sensitive to salt (842 proteins). The strongest salinity effect was seen at 31 °C, yielding both the highest proportion of affected proteins (1715 out of 2,440, 80.0%) and the largest median difference (0.644) (Supplementary Table S2). Conversely, temperature effects were most pronounced at 3% S; indeed, the increase from 20 °C to 31 °C and, moreover, from 31 °C *vs* 37 °C, affected roughly 79% of proteins. Thus, higher salt levels appear to sharpen the bacterium’s temperature response. Nevertheless, the specific response to salinity and temperature changes ultimately depended on the nature of the individual protein. For example, two chaperonins groEL2 and groEL1 were found among the most extreme interaction proteins affected, groEL2 expression rose by 4.28 units between 20 °C and 31 °C at 3% S, one of the largest single shifts in the dataset. The number and trends of the differentially expressed proteins (DEPs) detected in each pairwise comparison are shown in Figure 1B. Also, an interactive explorer for all 2,144 proteins with significant salinity **×** temperature interactions can be found in Supplementary material.

The largest response was found at 31 °C between both salinity 3% *vs* 1% conditions, with 678 differential proteins. Also, the strongest temperature-specific comparison was seen at 3% S, between 37 °C *vs* 31 °C, with 620 DEPs. Furthermore, while protein changes at 1% S were largely unidirectional and generally upward, trends at 3% salinity were temperature dependent. Further analysis of the 2,144 salinity x temperature interacting proteins revealed that between 83.2 and 93.5% of DEPs in any given comparisons were altered in response to a specific salt and heat combination rather than a uniform single-factor response (Figure 1C). The two largest overlaps included 580 of 620 proteins altered for the 3% S with 37 °C *vs* 31 °C comparison, and 623 out of 678 proteins in the 3% *vs* 1% S comparison at 31 °C.

In agreement with these results, the PCA analysis demonstrated a clear separation between salinity and temperature conditions alongside high replicate reproducibility (Figure 1D), indicating that each salt-and-heat combination promotes a distinctive proteomic profile in *Vibrio parahaemolyticus* rather than an additive sum of the two separated factors.

### 3.2 Proteome expression trends across temperature and salinity

Proteins were grouped into six clusters, based on their response trends across the six salinity–temperature conditions (Figure 1E; Supplementary Table S3). These findings corroborated that the proteome did not respond uniformly to temperature or salinity. Instead, distinct groups of proteins displayed contrasting expression trajectories, supporting the strong salinity × temperature interaction reported. Thus, in the largest group, Cluster 5 (*n* = 534 proteins), protein levels were low under 1% S but their expression preferentially increased under the higher salinity and temperature (3%S/37 °C) condition. Conversely, Cluster 1 (*n* = 453) showed the opposite pattern, with proteins relatively abundant at 1% S, especially at 20 °C and 31 °C, but their levels decreased under 3% S, mainly at 37 °C, suggesting repression or reduced accumulation under the most stressful combined condition.

Other clusters exhibited non-linear responses at intermediate temperatures: Cluster 4 (*n* = 313) and Cluster 2 (*n* = 369) both peaked at 3% S/31 °C before sharply declining at 37 °C. Cluster 3 (*n* = 440) increased with temperature (20 °C to 37 °C) at 1% S, but dropped markedly at 3% S/31 °C. Finally, Cluster 6 (*n* = 511) displayed a complex trajectory, increasing at 1%/31 °C and 3% S/37 °C, but dipping at 3% S/31 °C.

Overall, our findings demonstrate that the proteome of *Vibrio parahaemolyticus* is dynamically, non-linearly modulated, in response to environmental fluctuations of both, salinity and temperature factors.

### 3.3 Subcellular distribution of *Vibrio parahaemolyticus* altered proteins in response to salinity and temperature fluctuations

Further characterization of the DEPs revealed that most altered proteins were localized in the cytoplasm (36.8%) or classified within other cellular compartments (51.6%), whereas smaller proportions corresponded to periplasmic (4.9%), outer membrane (4.2%), inner membrane (1.9%), and extracellular proteins (0.6%) (Figure 2A). This distribution suggests that environmental fluctuations primarily affect intracellular metabolic and regulatory pathways, alongside membrane-associated adaptive responses. Across all six clusters, annotated proteins were predominantly cytoplasmic, whereas inner-membrane proteins represented the second most common compartment (Figure 2B). Notably, Cluster 4 exhibited the strongest membrane-associated profile, with equal cytoplasmic and inner-membrane proteins representation, consistent with its enrichment in respiratory and oxidative-phosphorylation functions. Clusters 2 and 5 also showed a relatively greater proportion of inner-membrane proteins. Nevertheless, only 18–20% of proteins per cluster had database-supported subcellular-location annotations, therefore these results should be interpreted as describing the annotated subset of the proteome.

**Figure 2:**
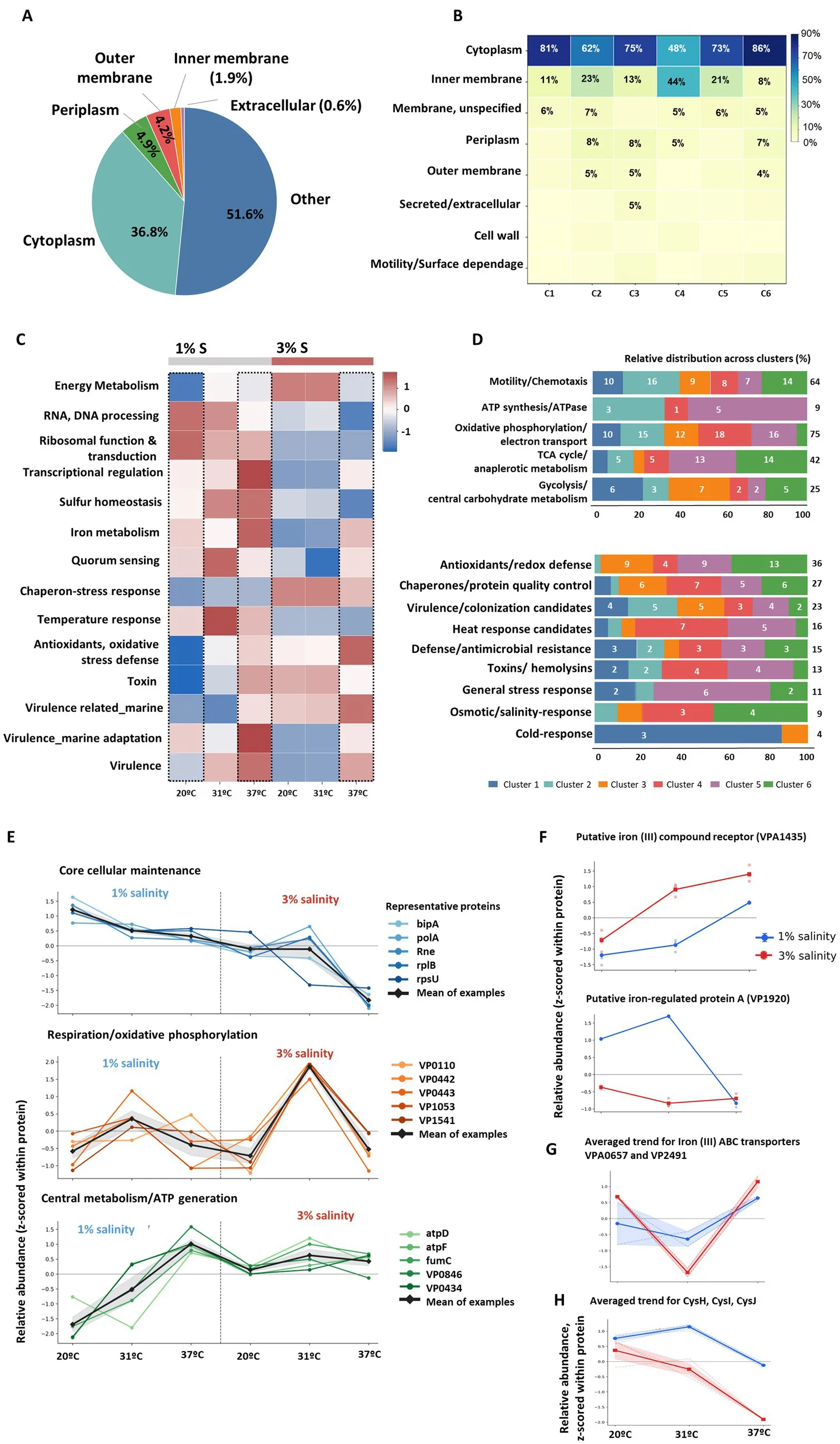
Functional classification and adaptive proteomic responses of Vibrio parahaemolyticus to environmental fluctuations. **(A)** Subcellular localization distribution of differentially expressed proteins (DEPs) identified under varying salinity and temperature conditions. **(B)** Subcellular localization distribution of DEPs across expression clusters. **(C)** Heatmap representing functional categories of DEPs associated with metabolism, transcriptional regulation, stress response, oxidative defense, quorum sensing, and virulence under different salinity (1% and 3%) and temperature (20 °C, 31 °C, and 37 °C) conditions. Red and blue colors indicate relative upregulation and downregulation, respectively. **(D)** Relative distribution of functions and total number of proteins assigned per cluster. **(E)** Expression trend graphs of representative proteins linked to core cellular maintenance, respiration/oxidative phosphorylation and central metabolism/ATP generation across 1%S and 3%S and temperatures 20 °C, 31 °C, 37 °C. **(F)** Individual expression trend lines of representative iron intake. **(G)** Averaged trend line graphs for iron (III) ABC transporters and **(H)** sulfur assimilation/metabolism proteins.

### 3.4 Metabolic and core cellular-maintenance reprogramming in *Vibrio parahaemolyticus* under salinity and temperature fluctuations

Functional analysis of DEPs revealed extensive and condition-dependent proteomic reprogramming in *Vibrio parahaemolyticus* in response to salinity and temperature interactions. DEPs spanned central metabolism and core cellular-maintenance processes as well as several stress-response networks, and virulence-associated mechanisms (Figures 2C, 3), which were widely distributed across the clusters (Figure 2D).

**Figure 3:**
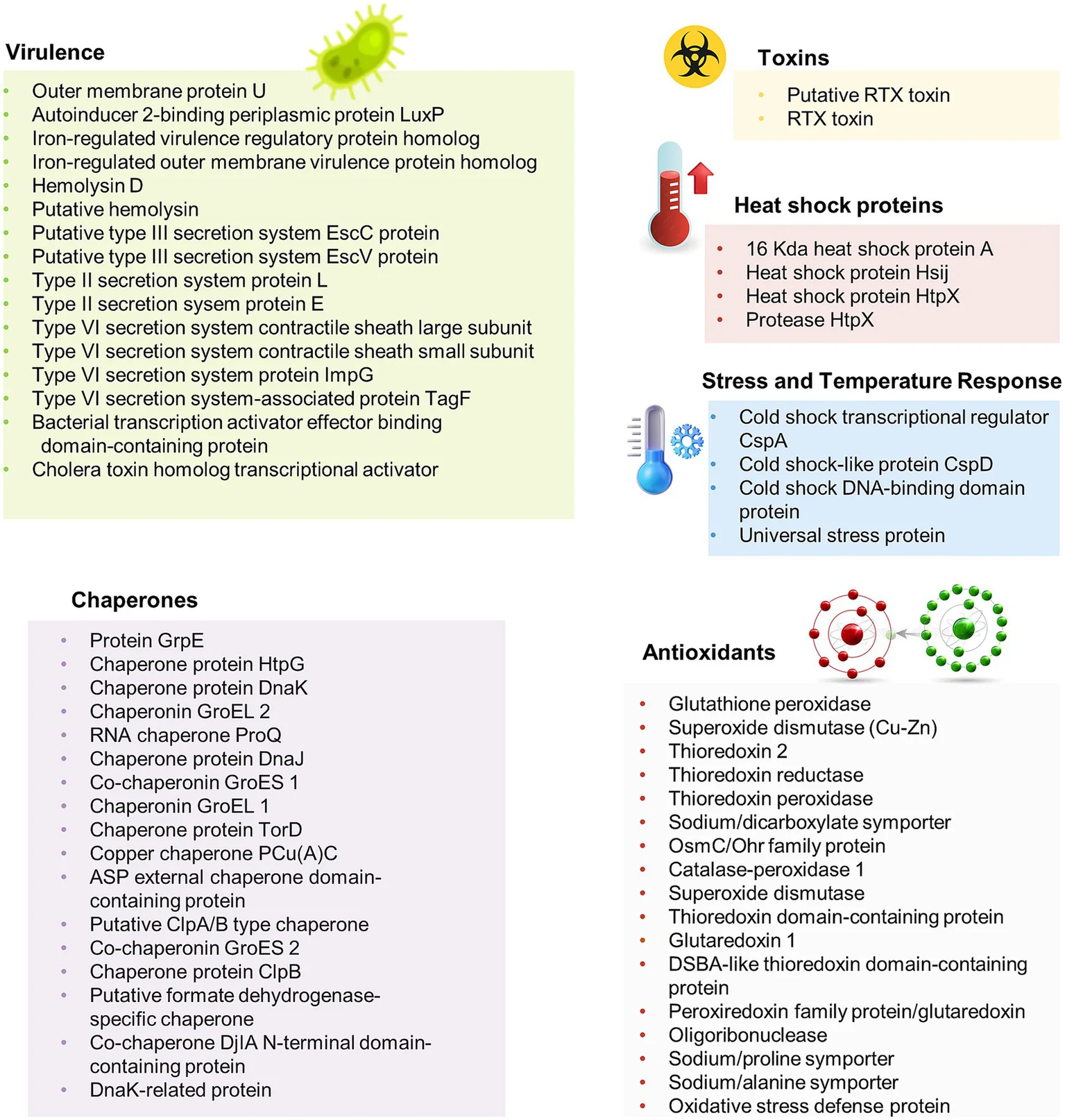
Main factors associated with virulence, stress response, and adaptive mechanisms identified in the studied microorganism. The figure highlights proteins and systems related to virulence, including outer membrane proteins, type II, III, and VI secretion systems, hemolysins, and transcriptional regulators. In addition, proteins involved in toxin production, heat shock response, stress and temperature response, molecular chaperones, and antioxidant enzymes associated with oxidative stress defense are shown. These elements suggest a strong capacity for adaptation and survival under adverse environmental conditions and during host interaction.

Core cellular-maintenance proteins, including translation/ribosome-associated proteins, RNA-processing factors and DNA replication/repair proteins (e.g., DNA polymerase I (polA), ribonuclease E (rne)), showed highly significant condition-specific contrasts and significant salinity × temperature interactions (Figure 2E). Specifically, proteins like the ribosomal assembly factor bipA, ribosomal proteins rplB and rpsU, included in cluster 1, were substantially downregulated at 37 °C under 3%S relative to 1% S. Similarly, energy metabolism-related proteins, including glycolysis and central carbohydrate metabolism, tricarboxylic acid cycle (TCA) and anaplerotic reactions, oxidative phosphorylation, electron transport, and ATP synthesis (e.g., AtpE, AckA2, FumC, NqrE, NapA, and PhnX), displayed marked condition-dependent expression changes. In general, their expression was relatively low at 1% S and 20–31 °C, increased at 1% S and 37 °C, and reached their highest average relative abundance at 3% S and 31 °C. Their average profile subsequently declined at 3% salinity and 37 °C. Alternatively, other proteins linked to central carbohydrate metabolism (including key glycolytic enzymes), represented in Cluster 3, showed increased expression with temperature at 1% S but downregulation at 3% S and 31 °C. This glycolysis-associated category did not reach statistically significant enrichment; therefore, it should be considered as a cluster-associated functional trend rather than a pathway-level enrichment.

Respiratory remodeling was most clearly evidenced by Cluster 4, containing 18 oxidative-phosphorylation/electron-transport candidates, including cytochrome proteins, ubiquinol-cytochrome *c* reductase components, succinate-dehydrogenase membrane-anchor proteins, and terminal oxidase components. These proteins were mostly upregulated at 3% S and 31 °C. KEGG analysis confirmed a significant enrichment of oxidative phosphorylation in this cluster (8 mapped proteins, FDR = 0.0168), supporting increased respiratory and ATP-generating capacity at 3% S and 31 °C, potentially supplying the energy required for ion homeostasis, membrane transport, protein repair, and stress adaptation.

Other proteins associated with TCA-cycle/anaplerotic-metabolism, ATP-synthesis/ATPase and oxidative-phosphorylation/electron-transport were included in cluster 5. For example, ATP synthase subunit atpF, fumarate hydratase fumC, succinate-dehydrogenase components, and cytochrome *d* ubiquinol oxidase subunit III increased at 1% S and 37 °C relative to 20 °C, whereas the ATP synthase subunit atpD was increased at 3% S relative to 1% S at 31 °C. Finally, proteins involved in amino acid biosynthesis, lipid metabolism, and nucleotide metabolism displayed more variable, condition-dependent regulation. Generally, intermediate temperatures (31 °C), triggered a downregulation of several metabolic proteins, suggesting a lower metabolic investment under moderate, less stressful conditions.

Overall, these results indicate that salinity and temperature fluctuations drive a shift from growth-associated core cellular functions toward condition-specific metabolic, respiratory, and stress-adaptation programs, with 3% S/ 31 °C representing an active respiratory-remodeling state and 3% S/ 37 °C reflecting a more restrictive stress-survival condition.

### 3.5 Iron acquisition, storage, and sulfate/sulfite assimilation responses

A subset of proteins related with iron and sulfate/sulfite metabolism were also identified. Based on the heterogeneous profiles seen for iron-related proteins across temperature-salinity conditions, iron acquisition, transport, storage, and regulatory functions appeared to respond distinctively rather than as a single coordinated network (Figure 2F). The levels of the iron (III) receptor VPA1435 increased progressively with temperature, peaking at 3% S/37 °C. In contrast, putative iron-regulated protein A VP1920 was highest at 1% S (20–31 °C) and declined at 1%S/37 °C, reaching the baseline levels seen across all 3% S conditions. Meanwhile, iron (III) ABC transporter periplasmic binding proteins VPA0657 and VP249 were highly correlated (*r* = 0.82), reporting low abundance at 3% S/31 °C while an strong upregulation at 3% S/37 °C.

Unlike iron-associated proteins, sulfate- and sulfite-assimilation proteins exhibited a highly coherent response, showing a coordinated down-regulation under high-salinity and high-temperature stress (Figure 2G). Specifically, the levels of sulfite reductase components (CysJ, CysI) and phosphoadenosine 5′-phosphosulfate reductase (CysH), and sulfate adenylyltransferase subunits (CysN, CysD), showed high abundance at 1% S (20–31 °C) but strong repression at 3% S/37 °C. Adenylyl-sulfate kinase (CysC) peaked at 1% S/37 °C but was similarly reduced under high salinity (3% S, 31–37 °C). Overall, while iron homeostasis showed divergent, condition-specific shifts, assimilatory sulfur metabolism was coordinately repressed under combined high-salinity and high-temperature stress.

Finally, several TonB energy-transducing and transport components (VPA0155-TonB, VPA0154-ExbD2, VPA0151-TonB biopolymer transport component, and VPA0424- ExbD1), appeared upregulated under warmer conditions, particularly at 3% S and 31–37 °C. In contrast, TonB-dependent receptors (VP0168, VP0167, VP0164, and VP0163) were more abundant at 1% S and 37 °C or 3% S and 20 °C, but decreased at 3% S and 31 °C. Remarkably, TonB-proteins mediate, among others, outer-membrane transport, iron and nutrient uptake, or virulence-associated iron scavenging, being important for obtaining iron and other scarce nutrients during environmental adaptation (Kuehl and Crosa, 2010).

### 3.6 Motility changes in *Vibrio parahaemolyticus* under salinity and temperature shifts

Salinity and temperature shifts promoted substantial condition-dependent changes in the proteomic profile of *Vibrio parahaemolyticus* ST36, with pronounced effects over proteins associated with motility, stress-tolerance and environmental sensing proteins (Figure 4). Structural flagellins, including flaA, flaB/flaD, flaF, and flaG, peaked at 3% S and 20 °C, but their levels dropped markedly at 3% S and 31 °C, followed by a partial recovery at 37 °C. At 1% S, flagellin levels were stable, with only small changes across temperature (Figure 4A). Conversely, Flagellar-regulatory proteins such as the negative regulator of flagellin synthesis VPA0262, flagellar regulators FlhF and FlhG, and flagellar motor-switch protein FliM, reported relatively low levels at 1% S and 20–31 °C, but increased strongly at 1% S and 37 °C. Under 3% S, these regulators showed a more moderate response, with slightly higher relative abundance at 20–31 °C and a decrease at 37 °C. The divergence between structural flagellins and their regulators may indicate that flagellar assembly and flagellar regulation are not necessarily synchronized under salinity and temperature fluctuations.

**Figure 4:**
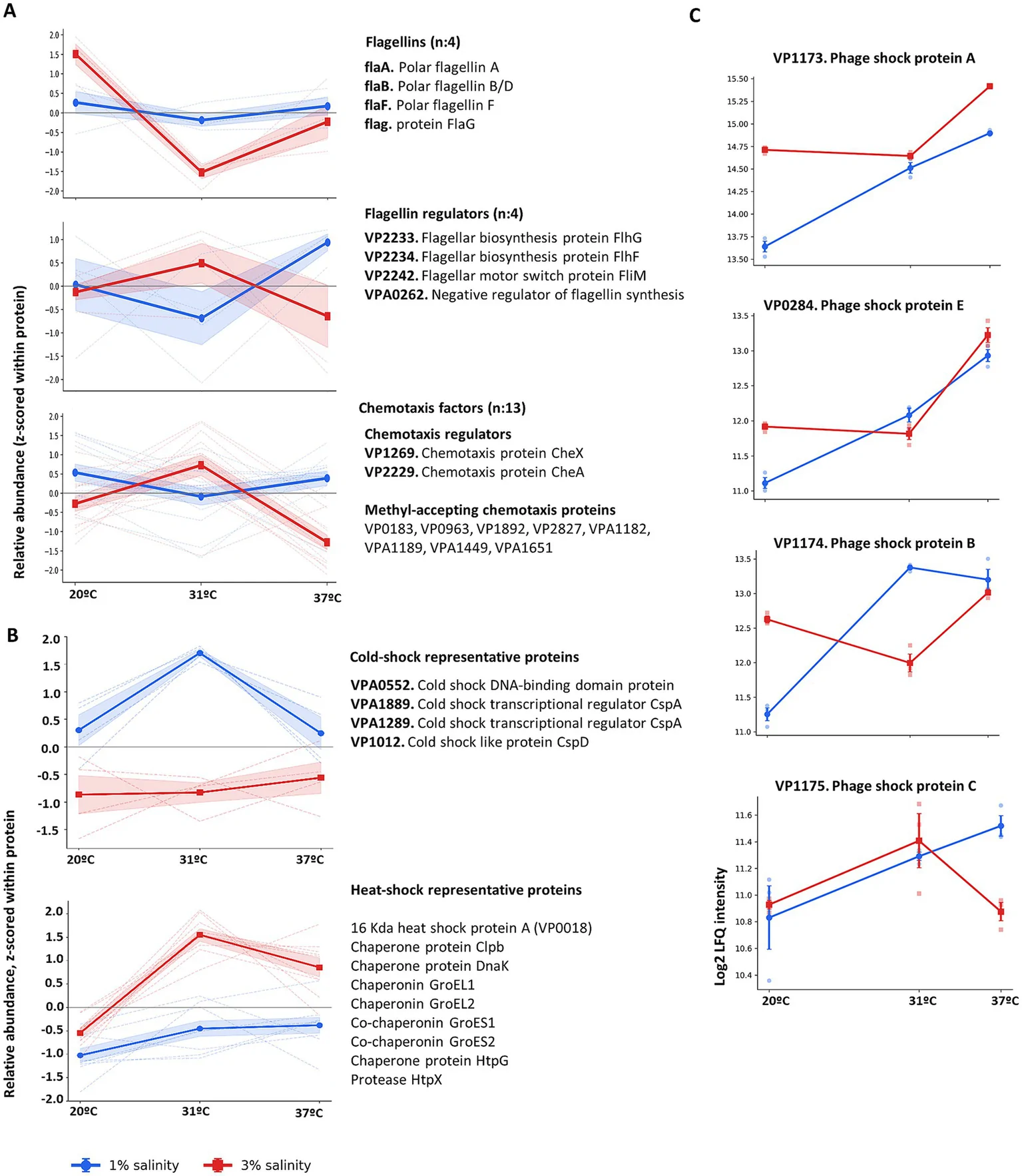
Differential expression of motility, chemotaxis, thermal-response and phage-shock proteins in *Vibrio parahaemolyticus* under salinity and temperature stress. **(A)** Average relative abundance profiles of flagellins, flagellin regulators and chemotaxis factors and **(B)** cold- and heat-shock proteins in *Vibrio parahaemolyticus* cultured under different temperature (20 °C, 31 °C, and 37 °C) and salinity (1 and 3% NaCl) conditions. Relative abundance (z-scored within protein) vs. Temperatures are shown. Faint dashed lines indicate individual protein profiles; bold lines and shaded regions represent group mean± SEM. **(C)** Expression trend lines for individual phage shock proteins. Protein expression values (profiles (Log2 LFQ intensity) are represented as log intensity means ± SEM (*n* = 3 biological replicates per condition). Statistical significance was evaluated using a two-way ANOVA followed by Bonferroni post-hoc test (*p* < 0.05).

On the other hand, many motility/chemotaxis proteins like Chemotaxis proteins CheA, CheX, and methyl-accepting chemotaxis proteins, showed relatively high abundance at 1% S/ 20 °C and at 3% S/31 °C, but declined at 3% S/37 °C. This suggests that intermediate high-salinity conditions may promote regulatory and motility-associated remodeling, whereas more severe temperature stress may reduce investment in energetically costly motility functions.

### 3.7 Thermal adaptation and stress response to temperature and osmotic changes

Thermal-adaptation proteins showed contrasting responses depending on salinity: Cold shock-like protein CspD, (VP1012); Cold shock transcriptional regulators CspA (VP1889, VP1289) and Cold shock DNA-binding domain protein (VPA0552) peaked at 1% S/31 °C and were reduced across all 3% salinity conditions. Conversely, most heat-shock proteins (9 out of 16 proteins, including ClpB, DnaK, dnaJ, HtpG, GroEL1, GroEL2, GroES1, GroES2, and GrpE) increased strongly at 3% S/31 °C and remained relatively elevated at 3% S/37 °C. This pattern suggests that high salinity shifts the thermal-response program e away from low-salinity cold-associated functions and toward heat-stress adaptation.

Similarly, several phage shock proteins (A–E) exhibited a not uniform stress-responsive patterns across the salinity-temperature conditions (Figure 4C). PspA and PspE levels increased with temperature at 1% S, although the strongest upregulation was seen at 3%S/37 °C. PspB, on the other hand, responded strongly to temperature under 1% S, peaking at 31 °C. Finally, PspC levels increased progressively with temperature under both 1 and 3%S, although the levels dropped again at 3%S/37 °C. These trends suggest that the Psp system may contribute to envelope or membrane-stress protection under adverse environmental conditions, although further assays should demonstrate the functional activation.

### 3.8 Virulence regulation and pathogenicity factors

Temperature and salinity shifts also modulated regulatory, toxins and virulence-associated proteins potentially involved in host interaction and pathogenicity (Figure 5A). Among them, the cholera toxin homolog transcriptional regulator (ToxR) was markedly upregulated at 1% 37 °C, whereas elevated 3% salinity dampened this increase, with toxR levels remaining at intermediate values, relatively stable across temperatures. Similarly, the RTX toxin activator expression increased from 20 °C to 31 °C at both salinity levels but declined at 3% S/37 °C, while the highest RTX toxin levels were observed at 1% S and 31 °C.

**Figure 5:**
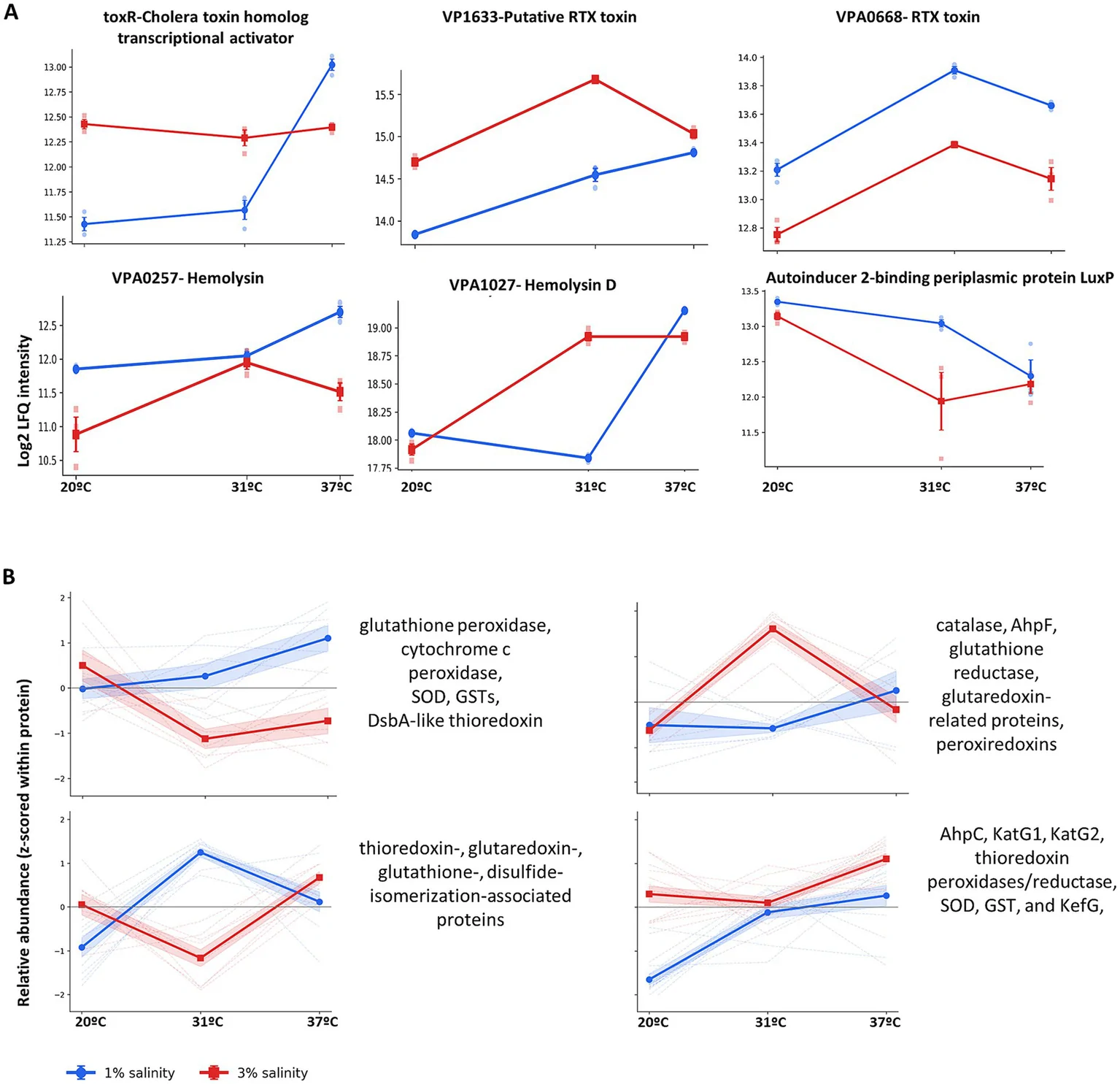
Temperature- and salinity-dependent regulation of virulence, pathogenicity, antioxidants and quorum sensing-associated proteins in *Vibrio parahaemolyticus*. **(A)** Expression profiles (Log2 LFQ intensity) of selected proteins involved in virulence regulation, pathogenicity, and quorum sensing in *Vibrio parahaemolyticus* under different temperature and salinity conditions. **(B)** Expression trends of antioxidant proteins categorized into four response groups showing an FDR-significant difference in at least one pairwise salinity or temperature comparison. Faint dashed lines show individual protein profiles; bold lines and shading show group mean± SEM. (n = 3 independent biological replicates per condition). Main effects and interactions were tested using a two-way ANOVA with Bonferroni’s post-hoc test (*p* < 0.05).

Although TDH or TRH were not directly identified in our study, in agreement with other studies evaluating *Vibrio parahaemolyticus* (Raghunath, 2014; Gutierrez West et al., 2013), several hemolysin isoforms were found together with ToxR and RTX toxins. Like RTX toxin, the hemolysin VPA0257 and the putative hemolysin VP2536, commonly associated with membrane disruption, cytotoxicity, nutrient release, or host-cell damage, reached their highest levels at 31 °C, decreasing at 37 °C, mostly at 3%S. A second group of isoforms, including Hemolysin D (VPA1027), hemolysin (VP2912), putative hemolysin (VP0446) together with putative RTX toxin (VP1633), showed a salinity-dependent behavior: at 3%S they followed similar pattern than the first group, increasing their expression at 31 °C and decreasing at 37 °C, but at 1%S the increase was even more pronounced at 37 °C. The distinct profiles detected suggest different regulatory pathways in response to temperature and salinity fluctuations, corroborating that virulence-associated proteins do not all peak under the same condition (Lovell, 2017).

### 3.9 Antioxidant defense, quorum sensing, and chaperone activity

Many antioxidant and redox-defense proteins were also identified as significantly altered in *Vibrio parahaemolyticus in* response to temperature and salinity changes. They were grouped in four main distinct condition-dependent trend groups (Figure 5B), including glutathione peroxidase, cytochrome c peroxidase, SOD, GSTs, and a DsbA-like thioredoxin, which increased exclusively at 1% S/37 °C but was suppressed under all 3% salinity conditions, indicating a low-salinity thermal defense program superseded by high salinity. Also, catalase, AhpF, glutathione reductase, glutaredoxin-related proteins, and peroxiredoxins peaked sharply at 3% S/31 °C before declining at 37 °C, representing a focused oxidative-detoxification response to intermediate osmotic stress. Trend Group 3 included nine thioredoxin-, glutaredoxin-, glutathione-, and disulfide-isomerization-associated proteins, which showed high abundance at 1% S/31 °C and dropped markedly at 3% salinity/31 °C, and recovered at 3%S/37 °C, reflecting dynamic structural reorganization of thiol-based buffering. Finally, the largest number of proteins, including AhpC, KatG1, KatG2, thioredoxin peroxidases/reductase, SOD, GST, and KefG, exhibited a progressive increase under high salinity that peaked at 3% S/37 °C, representing a comprehensive late-stage defense network covering peroxide detoxification, superoxide defense, thiol control, and ion homeostasis.

Overall, these distinct trends indicate that *Vibrio parahaemolyticus* employs condition-specific redox defense strategies rather than a single monolithic system. Intermediate stress (3% S/31 °C) triggers a focused catalase-, peroxiredoxin-, and glutathione-reductase response, whereas severe combined stress (3% S/37 °C) mobilizes the broadest protective network—integrating catalase-peroxidases, thioredoxin peroxidases, superoxide dismutase, and potassium-efflux machinery (*kefG*). This expanded response confirms that concurrent high salinity and elevated temperature generate an intensified oxidative and envelope-stress environment requiring a multifaceted proteomic defense.

Conversely, Quorum sensing-associated proteins, including the autoinducer-2 binding protein LuxP, also varied according to salinity and temperature, supporting the idea that environmental stress may influence bacterial communication and population-dependent regulation mechanisms. Indeed, LuxP levels decreased progressively with temperature at 1% salinity, and such downregulation was more pronounced at high salinity, specifically at 3%S/31 °C, where its levels were the lowest. This decline at higher salinity rates, suggesting that osmotic stress may affect cell-to-cell communication or environmental sensing.

## 4 Discussion

Our findings demonstrate that *Vibrio parahaemolyticus* ST36 exhibits a complex, non-linear and multifactorial proteomic response to concurrent variations in salinity and temperature, reflecting the physiological plasticity of this pathogen essential for its survival and infective capacity in fluctuating environments (Raghunath, 2014; Baker-Austin et al., 2018). Furthermore, these responses are not uniform but strongly dependent on the interaction of both, salinity and temperature, stressors.

A hallmark of this response is the extensive metabolic restructuring observed under environmental stress. Indeed, the upregulation seen of enzymes involved in the TCA cycle, amino acid metabolism, ATP synthesis and transport systems, suggest a substantial energetic investment to provide the energy and molecular precursors required to maintain homeostasis, support cellular defense mechanisms and virulence-associated processes under stressful environmental conditions, as previously described (Letchumanan et al., 2014; Fey et al., 2013). Concurrently, the relevant number of identified DEP related to iron acquisition, sulfur metabolism, and oxidative stress defense, supports the importance of redox balance and nutrient acquisition during environmental adaptation.

A compelling finding of this study was the strong condition-dependent proteomic reorganization observed for *Vibrio parahaemolyticus* ST36 in response to salinity and temperature shifts. Under relatively milder conditions, particularly at lower salinity, protein clusters were dominated by functions associated with core cellular maintenance, including translation, ribosome-associated processes, DNA replication and repair, and RNA turnover. These functions are consistent with an actively growing physiological state in which cells maintain protein synthesis, genome integrity, and routine metabolic activity. As salinity and temperature increased, particularly at 3% salinity and 31 °C, the proteome of *Vibrio parahaemolyticus* shifted toward an active metabolic-remodeling state required for environmental adaptation, characterized by an increase of respiratory proteins, oxidative phosphorylation components, central carbon-metabolism enzymes, ATP synthase subunits, chaperones, heat-response proteins, antioxidant defenses, membrane-associated proteins, and osmotic-stress candidates. Conversely, under the highest temperature and salinity condition (3% S/ 37 °C) a more restrictive stress-survival profile was seen, characterized by reduced abundance of core-maintenance and sulfate-assimilation proteins together with increased antioxidant, osmotic-stress, and envelope-protection responses. This last combination (3%/37ªC) appeared as the most physiologically demanding environment tested.

The divergent expression profiles of structural flagellins and flagellar regulatory proteins found in this study may be representative of a fundamental decoupling between structural filament accumulation and regulatory control during environmental fluctuations. While structural flagellins (*flaA*, *flaB/flaD*, *flaF*, *flaG*) peaked under hypersaline, low-temperature conditions (3% S/20 °C), key regulators—including the negative regulator VPA0262, spatial/numerical regulators FlhF and FlhG, and motor-switch protein FliM—were strongly upregulated under low salinity and high temperature (1% S/37 °C). This condition-dependent mismatch indicates that regulatory signaling does not linearly dictate structural filament assembly.

Virulence-, toxin-, motility-, chemotaxis-, and defense-associated candidates were distributed across several clusters rather than restricted to one condition. Specifically, key virulence factors such as ToxR, RTX toxins, several hemolysin isoforms and secretion system-associated proteins were mostly upregulated at 1% salinity and 37 °C, in agreement with previous studies reporting a positive correlation between elevated temperatures and increased pathogenic potential (Su and Liu, 2007; Letchumanan et al., 2014; Park et al., 2018) and higher incidence of seafood consumption outbreaks during warmer months (Baker-Austin et al., 2018). On the other hand, at 3% salinity, their highest increase occurred at 31 °C, followed by a downregulation at 37 °C, suggesting that temperature may modulate toxin-associated proteins in a salinity-dependent manner, rather than a simple linear increase under severe stress.

Hypersaline conditions (3%) promoted the overexpression of numerous proteins involved in defense mechanisms, including chaperones, antioxidant proteins and components of the type VI secretion system, in agreement with previous reports (Wong et al., 2000; Zhang and Orth, 2013; Caburlotto et al., 2010). Similarly, elevated salinity also affected the thermal-response network, by reducing the expression of cold-shock proteins (CspD, CspA, VPA0552), while increasing the presence of major heat-shock chaperones (ClpB, DnaK, DnaJ, htpG, GroEL1/2, GroES1/2, and GrpE), thereby prioritizing chaperones to mitigate severe protein misfolding and proteotoxicity under combined osmotic and thermal stress. Concurrently, the progressive downregulation of the quorum-sensing regulator LuxP at higher salinity and temperature rates may represent an adaptive strategy favoring cell motility, bacterial dispersal and environmental exploration under stressful conditions, rather than biofilm-associated community behavior, as previously described in other *Vibrio* species (Milton, 2006; Yildiz and Visick, 2009; Li et al., 2019).

Importantly, the present study differs substantially from previous proteomic investigations in both experimental design and biological scope. For instance, Guo et al. (2025) recently characterized the adaptation of *Vibrio parahaemolyticus* to dual stress conditions involving low salinity and bile salts, focusing mainly on survival and pathogenicity within the human intestinal environment. By application of targeted phenotypic assays (biofilm formation, motility assays, ROS production, and cytotoxicity experiments under low salinity and bile salt exposure), their work demonstrated alterations in motility, biofilm formation, secretion systems, and virulence-associated pathways under intestinal-like conditions. In contrast, based on a high-resolution proteomic quantitative approach, our work delivers an ecological perspective by charting the interplay between salinity and temperature, two central factors in marine ecosystems and seafood-associated habitats, and provides a broader ecological perspective by integrating how these stressors affect metabolic regulation, quorum sensing, oxidative stress defense, and virulence-associated proteomic remodeling. This workflow allowed us to identify coordinated proteomic responses that would be difficult to detect through targeted phenotypic analyses alone. Similarly, while previous temperature-focused proteomic and metabolomic studies mostly investigated metabolic and growth-associated changes under thermal stress (Meng et al., 2025), our results reveal that temperature-dependent responses are strongly modulated by salinity conditions.

Beyond the biological implications, the high-resolution LFQ-proteomics approach enabled a highly reproducible and sensitive quantification of stress- and virulence-associated proteins in a complex bacterial system. This approach not only provides a robust platform for studying environmental adaptation in *Vibrio parahaemolyticus* but also represents a scalable strategy for investigating proteomic responses in other marine pathogens under climate-related environmental changes.

Despite these comprehensive insights, two main limitations should be considered. First, because the study was performed using a single environmental ST36 isolate, the observed proteomic responses cannot be extrapolated to the entire ST36 lineage. Future multi-omics investigation, integrating genomics, transcriptomics and proteomic analyses across different geographical isolated strains will contribute to determine whether the adaptive responses observed here are conserved across pandemic *V. parahaemolyticus* lineages. Second, while the DEPs identified correlate with many of the aforementioned pathways, the altered protein abundance does not directly demonstrate functional activity or post-translational regulation; thus, future phenotypic and functional assays are required to confirm these physiological outcomes.

Taken together, our findings establish a foundation for future research to explore the precise role of the differential proteins identified while reaffirming temperature and salinity as critical environmental determinants of *Vibrio parahaemolyticus* ST36 physiology and pathogenicity. Crucially, the present work reveals that the interaction between these factors drives coordinated regulation of metabolic, virulence, stress-response, and signaling pathways. These proteomic alterations reflect potential adaptive mechanisms in this marine pathogen, suggesting that concurrent salinity and temperature shifts may directly influence survival, environmental persistence, and host-interaction capacity.

## Data Availability

The data underlying this article are available in the article and in its online Supplementary material. In addition, mass spectrometry proteomics data have been deposited to the ProteomeXchange Consortium via the PRIDE partner repository (Perez-Riverol et al., 2025) with the dataset identifier PXD080184.

## Footnotes

1
https://julius.ai/

## Supplementary material

Supplementary Table S1 *Vibrio* Two-way-anova results.

Supplementary Table S2 *Vibrio* interaction simple effects.

Supplementary Table S3 *Vibrio* protein expression trend clusters and functions.
