# Supplementary material for: Proteomic adaptation of the hypervirulent pandemic Vibrio parahaemolyticus ST36 lineage to salinity and temperature stress
Source: Front Microbiol. 2026 Oct 2;17:1947974. doi: 10.3389/fmicb.2026.1947974 (PMC13672198; doi:10.3389/fmicb.2026.1947974)
Supplement: Supplementary file 1 [file Supplementary_file_1.zip › reviewer_ready_interaction_explorer/salinity_temperature_interaction_trend_explorer.html]

## Salinity × temperature interaction protein trend explorer

Browse all 2,144 proteins with a significant interaction. Lines are condition means; faint points are the three technical replicates.

Interaction class

Search

Protein

The explorer is self-contained: it can be opened locally in a browser without internet access.
